# Supplementary material for: Phenanthroline-carbolong interface suppress chemical interactions with active layer enabling long-time stable organic solar cells
Source: Nat Commun. 2023 Jun 16;14:3571. doi: 10.1038/s41467-023-39223-9 (PMC10272153; doi:10.1038/s41467-023-39223-9)
Supplement: Supplementary file 1 — Supplementary Information [file 41467_2023_39223_MOESM1_ESM.pdf]

Phenanthroline-Carbolong Interface Suppress Chemical Interactions with Active Layer Enabling Long-Time Stable Organic Solar Cells

Xue *et al.*

SUPPLEMENTARY FIGURES AND TABLES

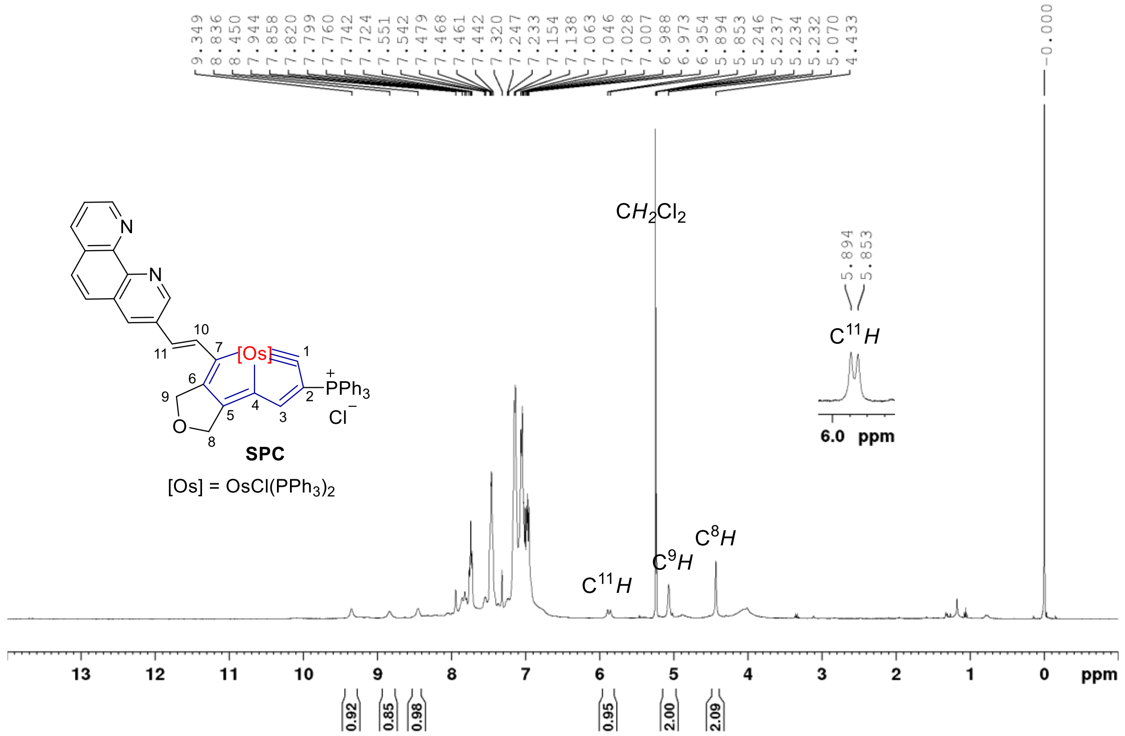

Supplementary Figure 1. The ^{1}H NMR (400.1 MHz, CD_2Cl_2) spectrum for SPC.

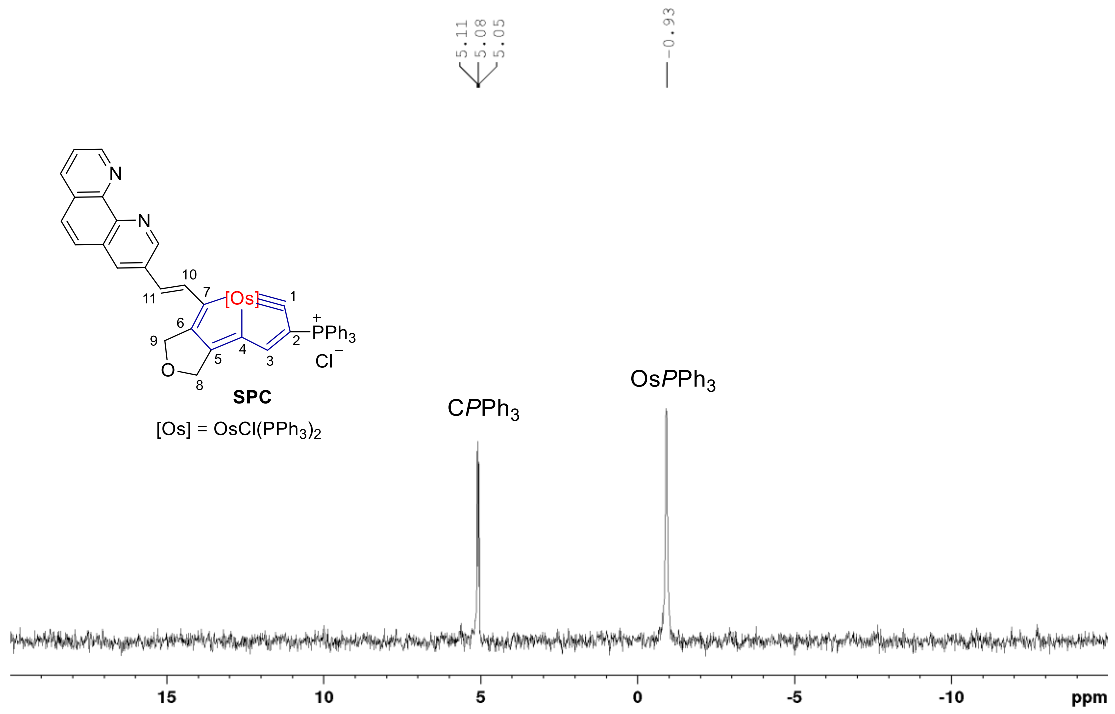

Supplementary Figure 2. The $^{31}P\{^{1}H\}$ NMR (162.0 MHz, CD_2Cl_2) spectrum for SPC.

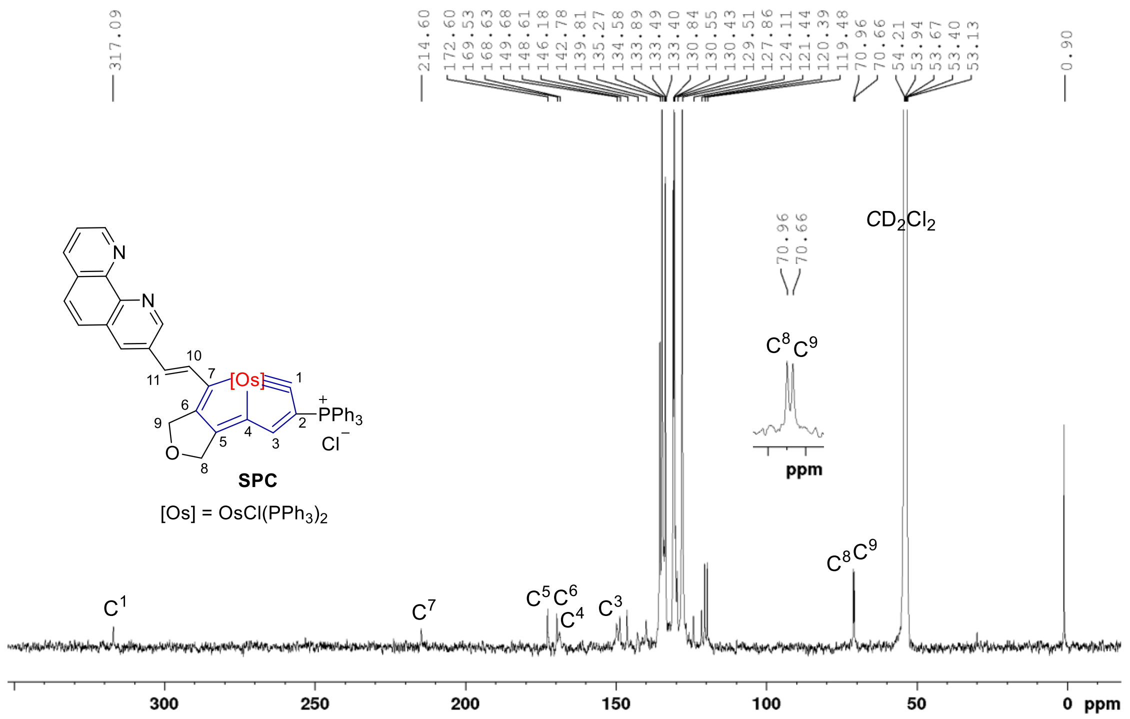

Supplementary Figure 3. The $^{13}C\{^{1}H\}$ NMR (100.6 MHz, CD_2Cl_2) spectrum for SPC.

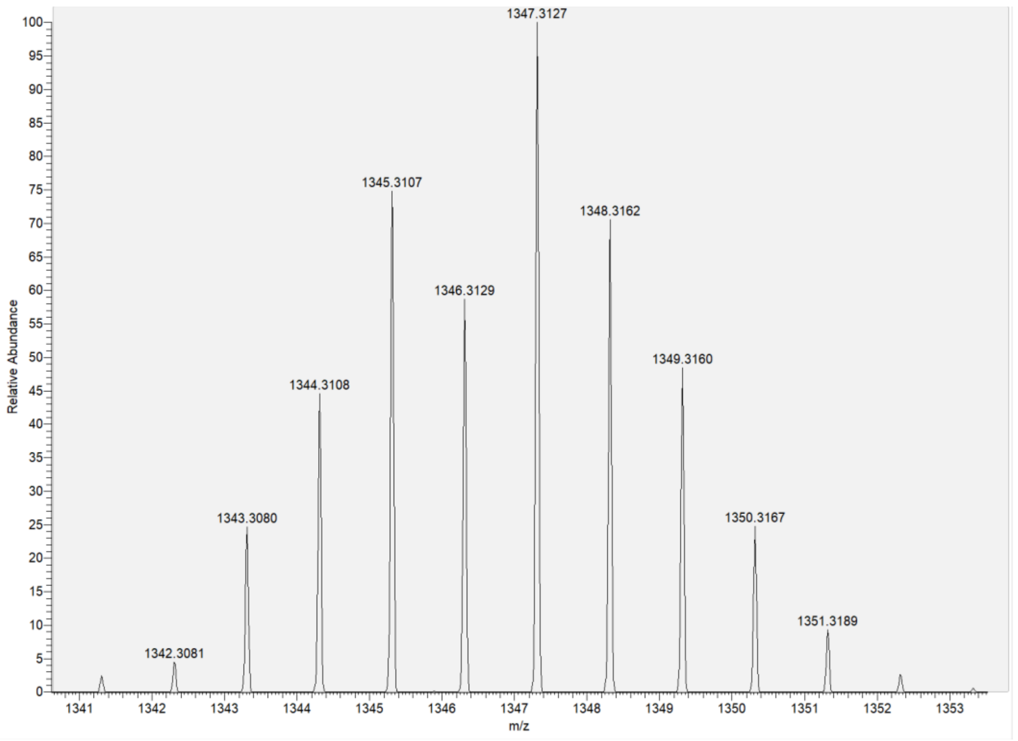

Supplementary Figure 4. Positive ion ESI-MS spectrum of [**SPC**]$^+$ measured in methanol.

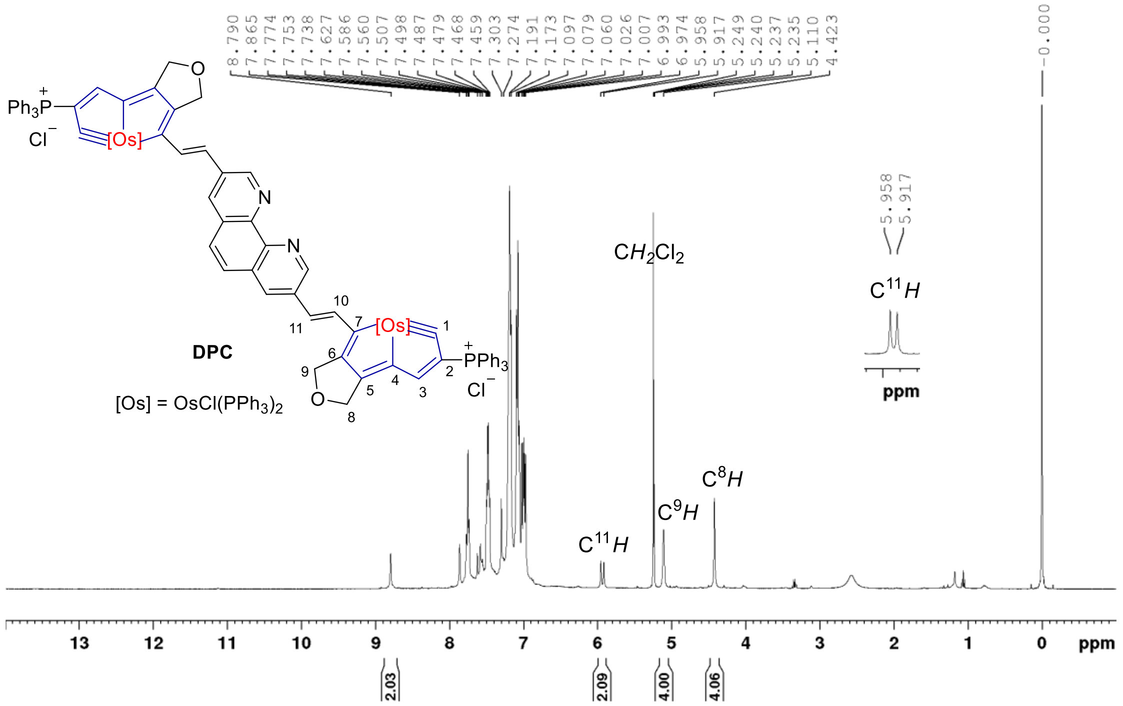

Supplementary Figure 5. The ^{1}H NMR (400.1 MHz, CD_2Cl_2) spectrum of DPC.

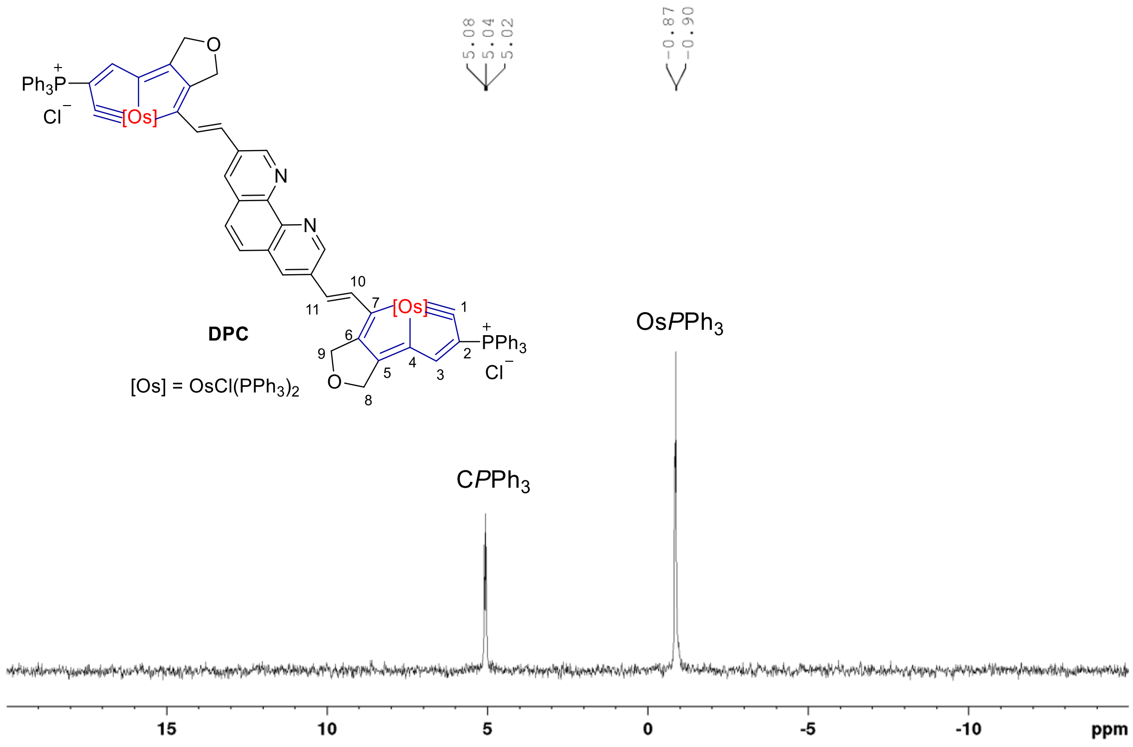

Supplementary Figure 6. The ^{31}P{^{1}H} NMR (162.0 MHz, CD_2Cl_2) spectrum of DPC.

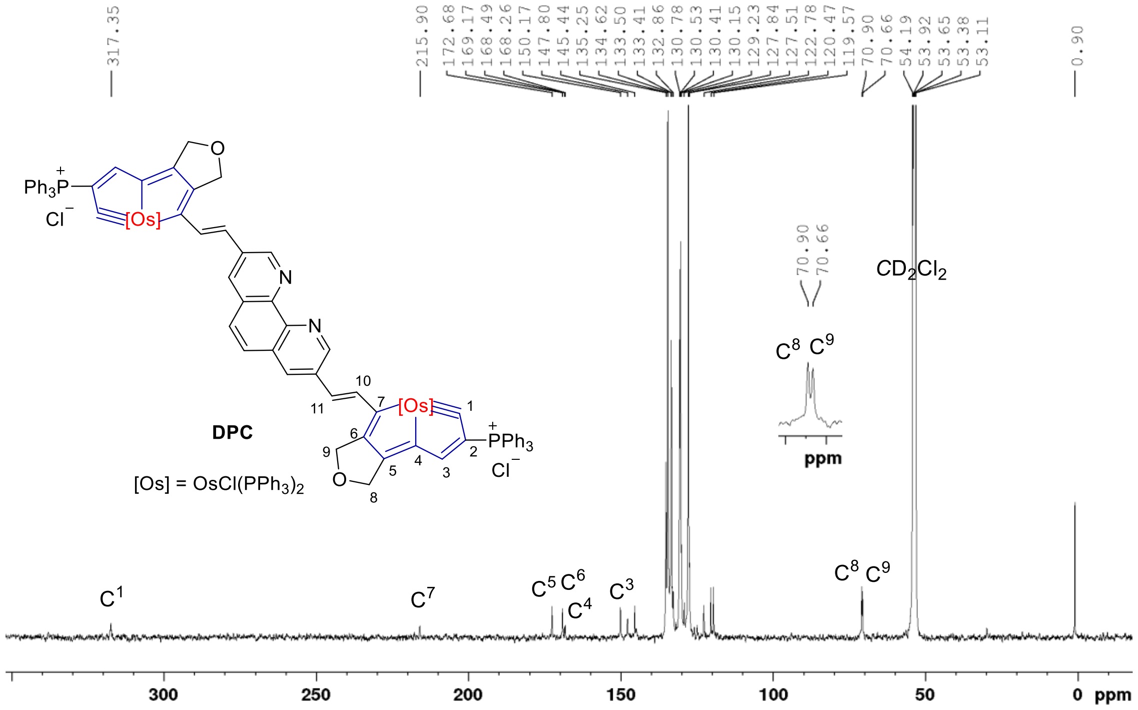

Supplementary Figure 7. The $^{13}C\{^1H\}$ NMR (100.6 MHz, CD_2Cl_2) spectrum of DPC.

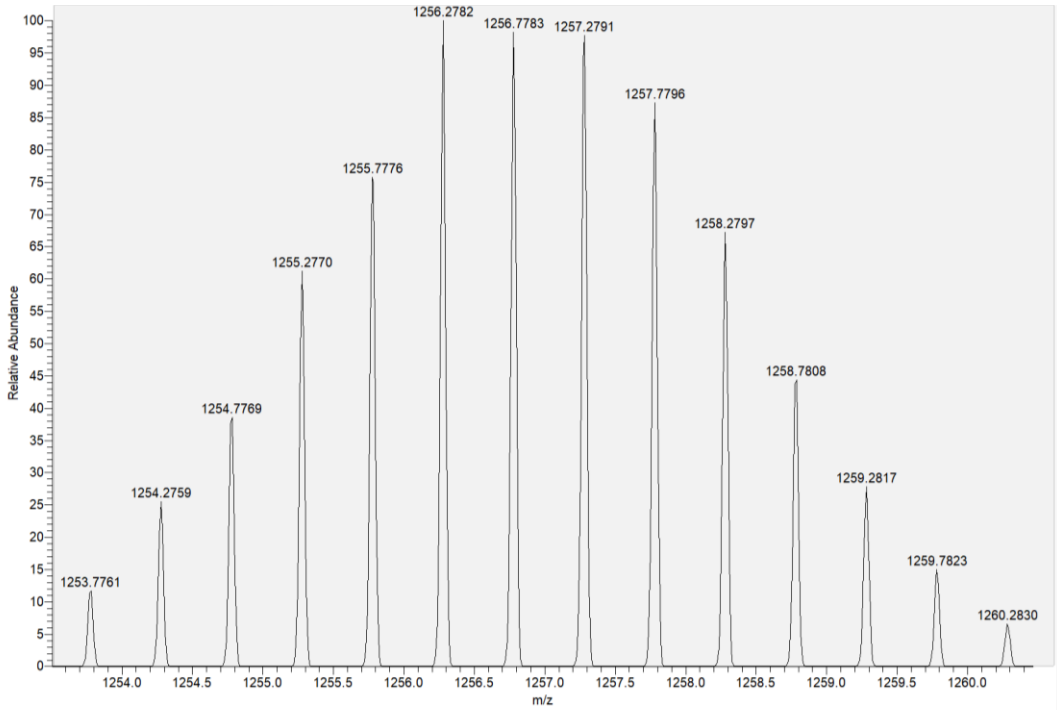

Supplementary Figure 8. Positive-ion ESI-MS spectrum of [**DPC**]$^{2+}$ measured in methanol.

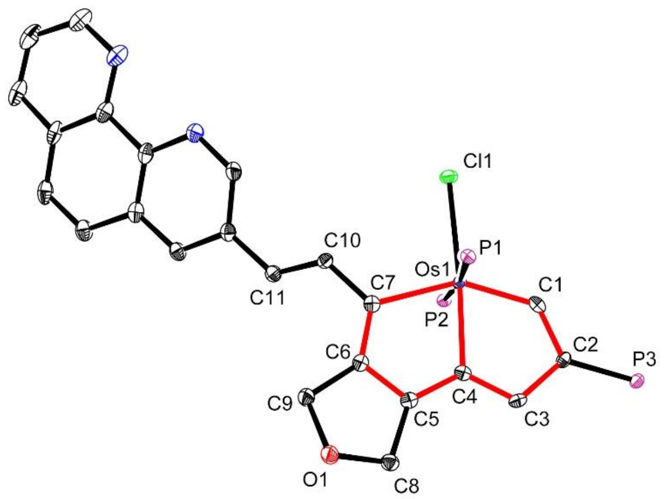

Supplementary Figure 9. X-ray molecular structure of the cation of complex **SPC** drawn with 50% probability level. The phenyl groups in PPh_3, aliphatic hydrocarbon and hydrogens are omitted for clarity. Selected bond lengths [Å] and angles [°]: Os1–C1 1.842(2), C2−C3 1.420(3), C6−C7 1.391(3), Os1–C4 2.102(2), C3−C4 1.388(3), C7–C10 1.455(3), Os1–C7 2.086(2), C4−C5 1.390(3), C10–C11 1.348(3), C1−C2 1.395(3), C5−C6 1.386(3). Os1–C1–C2 130.73(17), C4–C5–C6 115.1(2), C1–C2–C3 107.1(2), C5–C6–C7 116.1(2), C2–C3–C4 111.7(2), C6–C7–Os1 116.28(17), C3–C4–Os1 117.94(17), C7–Os1–C4 76.25(9), C4–Os1–C1 72.56(9), Os1–C7–C10 121.54(17), Os1–C4–C5 116.29(17), C7–C10–C11 123.2(2).

Supplementary Table 1. Crystal data and structure refinement of SPC.

	**SPC**
Empirical formula	$C_{78}H_{63}Cl_4N_2O_2OsP_3$
Mol. weight	1485.21
Temperature [K]	100.00
Crystal system	monoclinic
Space group	$P2_1/n$
a [Å]	12.6507(12)
b [Å]	16.1380(16)
c [Å]	32.033(3)
α [°]	90
β [°]	91.033(4)
γ [°]	90
V [Å^3]	6538.8(11)
Z	4
ρ_{calcd} [g cm^{-3}]	1.509
μ [mm^{-1}]	6.277
F(000)	3000.0
Crystal size [mm^3]	0.14 × 0.13 × 0.12
Radiation	CuKα (λ = 1.54178)
2θ range [°]	5.518 to 134.418
Coll. refl.	112178
Indep. refl.	11549
data/restraints/params	11549/0/824
GOF on F^2	1.121
R_1/wR_2 [$I \geq 2\sigma(I)$]	0.0240/0.0512
R_1/wR_2 (all data)	0.0249/0.0516
Largest peak/hole [e Å-3]	0.57/-0.73

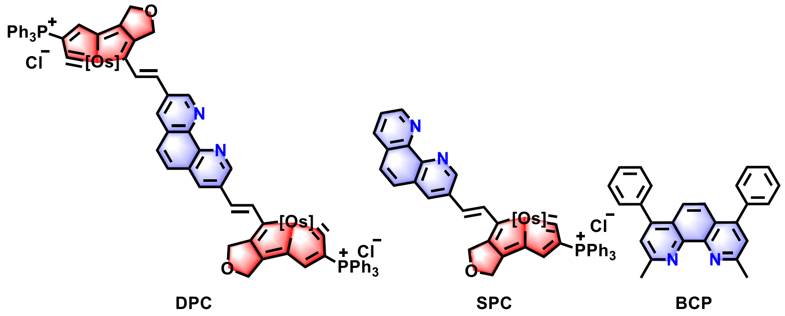

Supplementary Figure 10. The chemical structures of DPC, SPC and BCP.

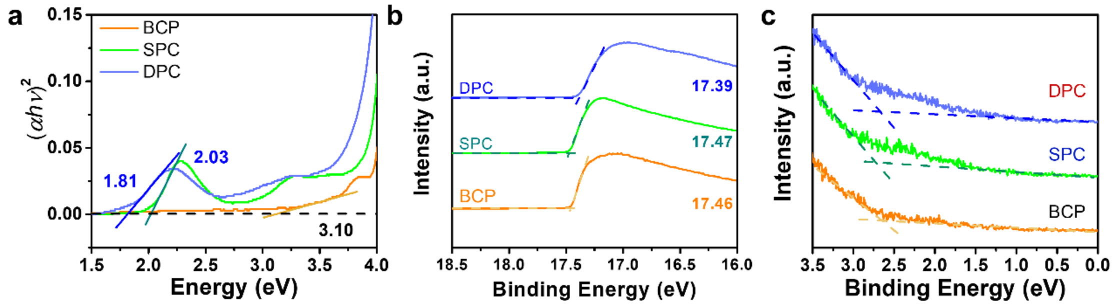

Supplementary Figure 11. (a) valence region of BCP, SPC, and DPC calculated from the absorption edge; UPS spectra of the film of BCP, SPC, and DPC (b) secondary electron cutoff and (c) valence region.

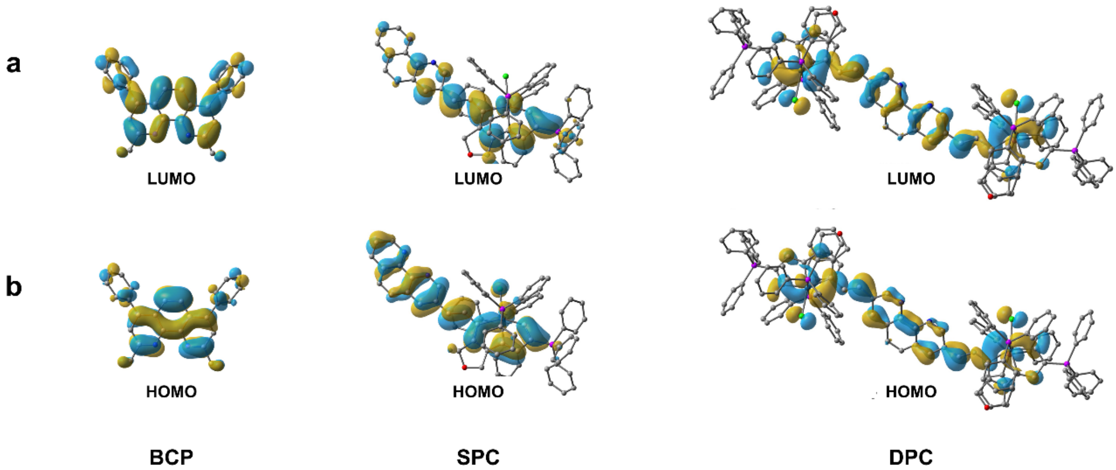

Supplementary Figure 12. DFT calculations of (a) LUMO energy level and (b) HOMO energy level of BCP, SPC and DPC (from left to right in each panel)

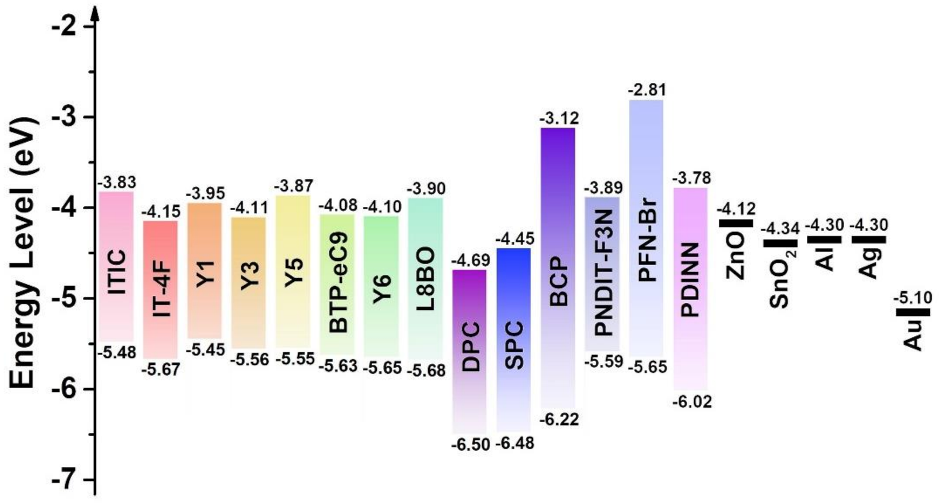

Supplementary Figure 13. Energy level of the NFAs, CIMs and electrodes that were used in high efficiency OSCs reported in recent years.

Supplementary Table 2. Energy level of the NFAs, CIMs and electrodes that were used in high efficiency OSCs reported in recent years.

Name	HOMO/VB (eV)	LUMO/CB (eV)	Reference
ITIC	-5.48	-3.83	[1]
IT-4F	-5.67	-4.15	[2]
Y1	-5.45	-3.95	[3]
Y3	-5.56	-4.11	[4]
Y5	-5.55	-3.87	[5]
BTP-eC9	-5.63	-4.08	[6]
Y6	-5.65	-4.10	[7]
L8-BO	-5.68	-3.90	[8]
DPC	-6.50	-4.69	This work
SPC	-6.48	-4.45	This work
BCP	-6.22	-3.12	This work
PNDIT-F3N	-5.59	-3.89	[9]
PFN-Br	-5.65	-2.81	[10]
PDINN	-6.02	-3.78	[11]
ZnO	/	-4.12	[12]
SnO_2	/	-4.34	[13]
Al	/	-4.30	[14]
Ag	/	-4.30	This work
Au	/	-5.10	[15]

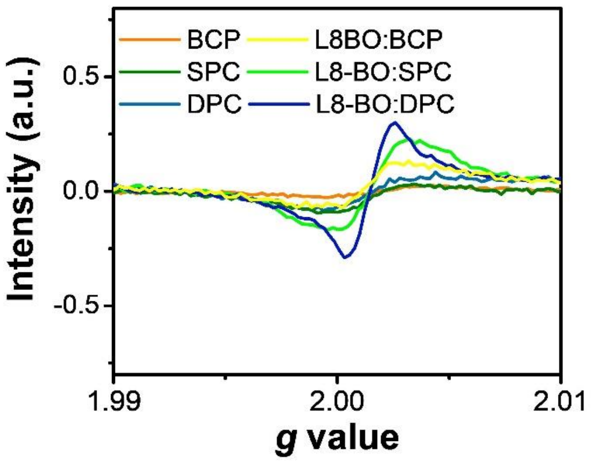

Supplementary Figure 14. ESR spectra of the solid samples of BCP, SPC, DPC, L8-BO:BCP, L8-BO:SPC and L8-BO:DPC in solid.

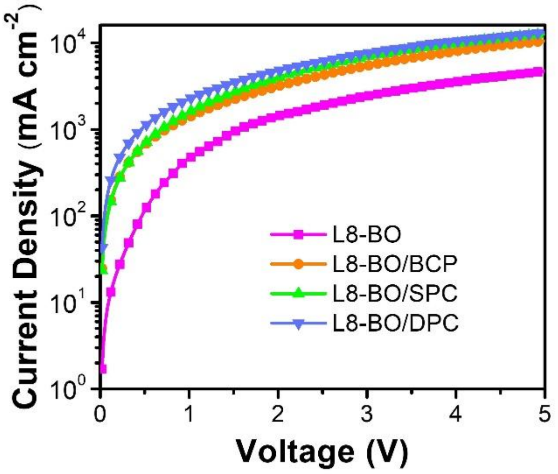

Supplementary Figure 15. The electron mobility of L8-BO covered with CIL of BCP, SPC and DPC or CIL free.

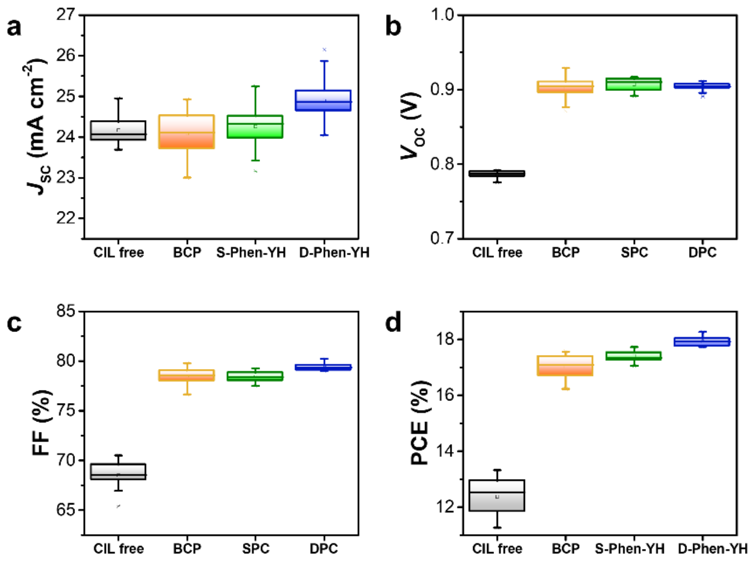

Supplementary Figure 16. (a) J_{SC}, (b) V_{OC}, (c) FF and (d) PCE distribution diagram of OSCs based on CIL free, BCP, SPC and DPC.

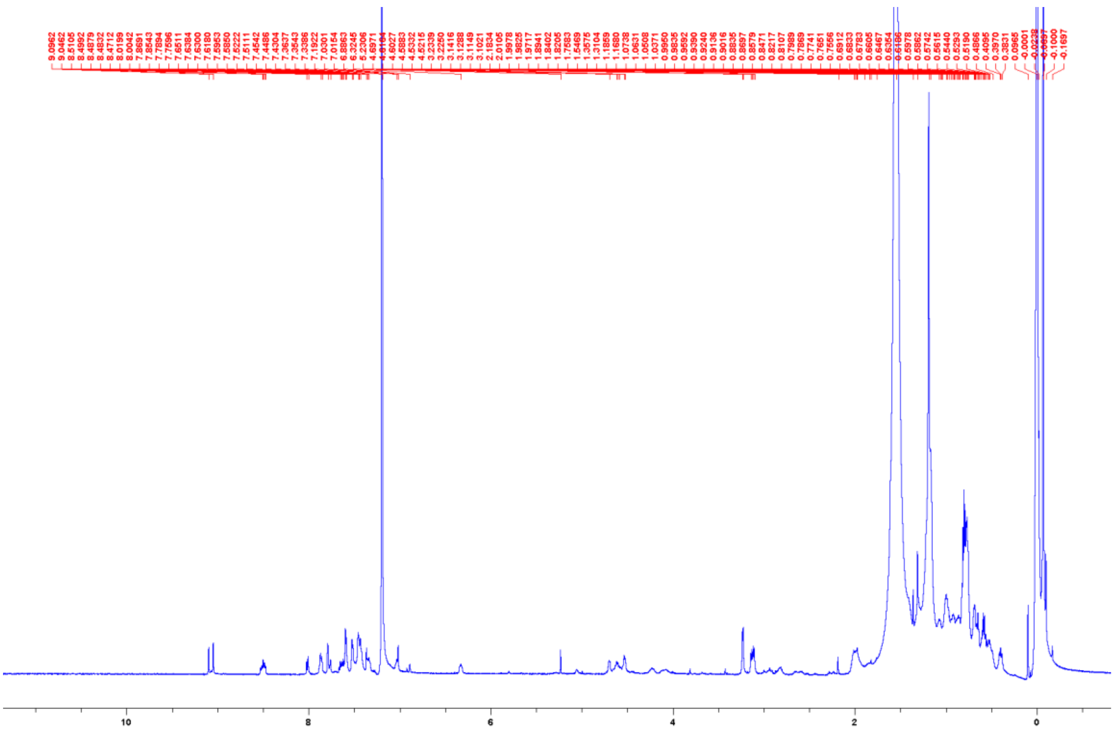

Supplementary Figure 17. The ^{1}HNMR spectra of the final product of BCP and L8-BO.

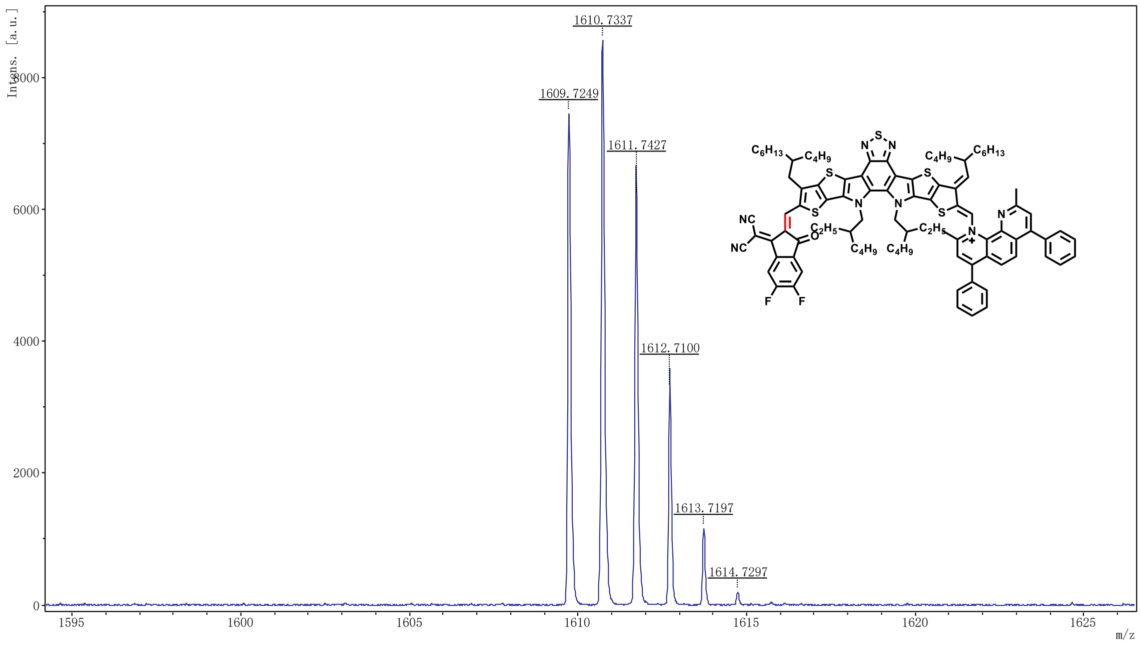

Supplementary Figure 18. The mass spectrum of the final product of BCP and L8-BO.

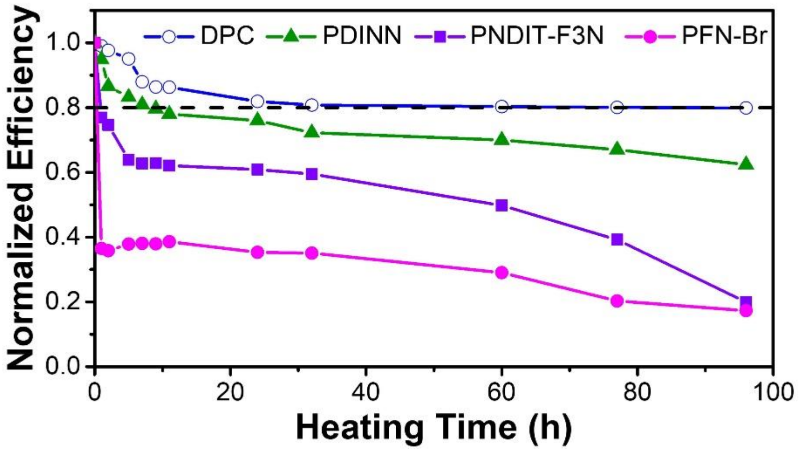

Supplementary Figure 19. The thermal stability (85 ^{o}C) of devices based on various CILs.

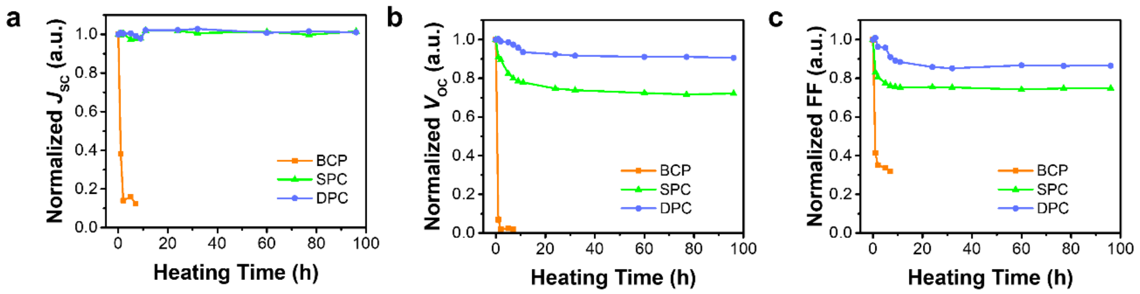

Supplementary Figure 20. The photovoltaic parameters change of device based on BCP, SPC and DPC CIL over heating time at 85 °C in N_2 atmosphere, (a) J_{SC}, (b) V_{OC} and (C) FF.

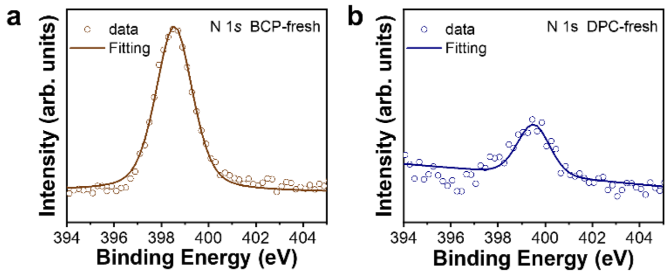

Supplementary Figure 21. The XPS spectra characterization of the signal from the N 1*s* orbital of (a) fresh BCP, (b) fresh DPC film

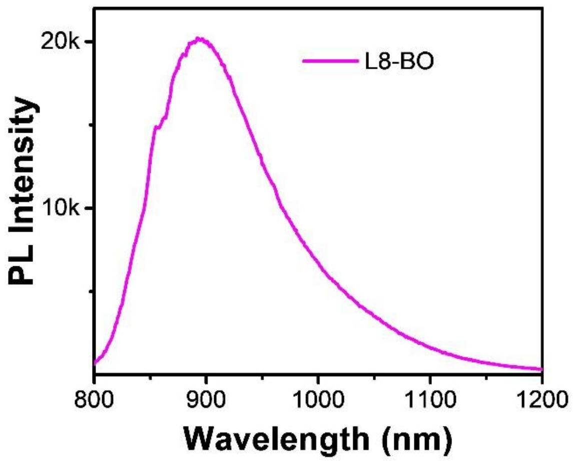

Supplementary Figure 22. The PL spectra of L8-BO film.

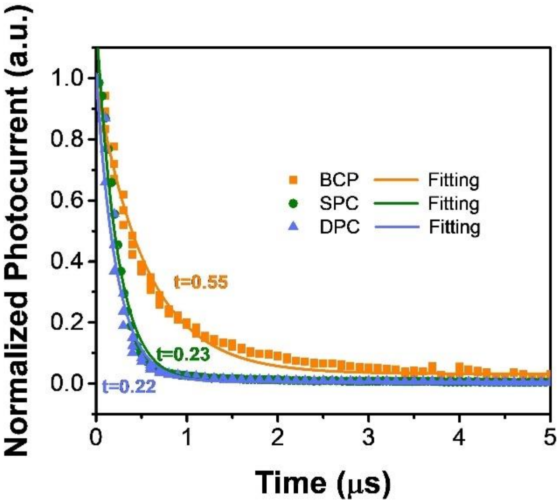

Supplementary Figure 23. TPC measurement of device based on different CILs.

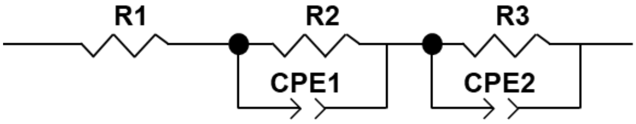

Supplementary Figure 24. Equivalent circuit diagram that be used for fitting the EIS spectroscopy.

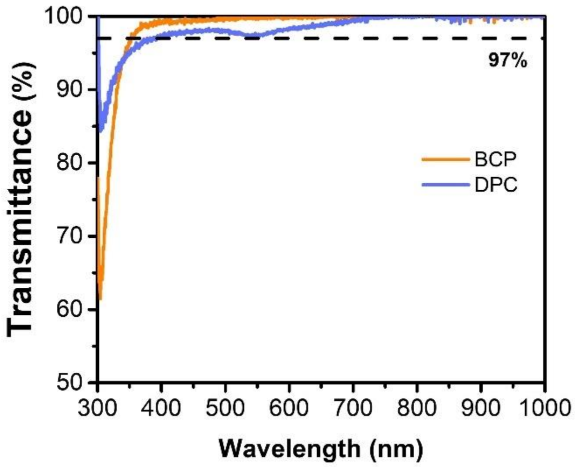

Supplementary Figure 25. The transmittance of optimized BCP and DPC film.

Supplementary Table 3. The concentration optimization of the DPC CIL for D18:L8-BO binary based OSC.

Concentration (mg/mL)	ICL	J_{SC} (mA cm^{-2})	V_{OC} (V)	FF (%)	PCE (%)
1	PDINN	25.48	0.891	79.1	18.0
0.5	PNDIT-F3N	24，92	0.906	78.1	17.6
0.5	PFN-Br	24.13	0.905	76.1	16.7
0.5	DPC	24.26	0.897	77.5	16.9
1		25.16	0.904	79.2	18.0
2		25.16	0.905	80.1	18.2
3		25.12	0.905	79.5	18.1
5		24.90	0.905	79.4	17.9
0.5	SPC	24.34	0.892	76.61	16.6
1		24.33	0.900	78.96	17.2
2		24.85	0.904	79.3	17.8
3		24.54	0.905	78.8	17.6
5		24.51	0.903	78.4	17.4

The best concentration of PDINN, PNDIT-F3N and PFN-Br can from previous reports [9,11].

Supplementary Table 4. Fitting parameters of EIS for BCP, SPC and DPC based device.

Device	R_1 (Ω)	R_2 (Ω)	R_3 (Ω)	CPE_{T1} (10^{-9} F)	$CPEP_1$	CPE_{T2} (10^{-9} F)	CPE_{P2}
BCP	16.6	31.6	616.1	10.9	0.9	0.6	1.0
SPC	21.4	25.7	833.5	12.8	0.9	6.2	1.0
DPC	9.9	364.7	3040.0	1.2	0.9	1.7	0.9

Supplementary Table 5. Photovoltaic parameters of the optional individual sub-cells and tandem solar cells.

Cells	ICM	Con. of ICM (mg/mL)	J_{SC} (mA cm^{-2})	V_{OC} (V)	FF (%)	PCE (%)
PVK	w/o	w/o	15.43	1.29	81.2	16.2
OSC	DPC	0	20.26	0.084	27.94	0.5
		0.5	21.90	0.854	62.36	11.7
		1	22.85	0.851	70.4	13.7
		2	21.90	0.853	58.71	11.0
		3	21.32	0.831	51.90	9.2
		5	21.07	0.819	43.71	7.5
	BCP	0.5	21.47	0.38	42.8	3.5
Tandem	DPC	1	12.95	2.07	80.8	21.7
Tandem	BCP	0.5	11.51	1.79	59.9	12.4

SUPPLEMENTARY METHODS

Synthesis of SPC

The carbolong complex S1 was synthesized according to published methods[16]. An excessive proton acid HCl • Et_2O (0.5 mL of a 2 M solution in ether) was added to a DCM solution (5 mL) of carbolong complex S1 (0.10 g, 0.085 mmol) and 3-ethynyl-1,10-phenanthroline Phen-S1 (0.019 g, 0.093 mmol) using standard Schlenk techniques. The reaction mixture was stirred at room temperature for 2 h to yield a violet solution. The solution was evaporated under vacuum to a volume of ~1 mL and then washed with Et_2O (1× 20 mL) to afford a violet solid. The solid was purified by flash chromatography on silica gel (eluent: 20:1 DCM/MeOH) to yield complex 1 as a violet solid. Yield: 0.11 g, 94%. ^{1}H-NMR (400.1 MHz, CD_2Cl_2): δ = 9.35 (s, 1H), 8.84 (s, 1H), 8.45 (s, 1H), 5.87 (d, J = 16.50 Hz, 1H, $C^{11}H$), 5.07 (s, 2H, C^9H), 4.43 (s, 2H, C^8H), 7.94-6.95 (51H, other aromatic protons,C^3H, and $C^{10}H$). ^{31}P-NMR (162.0 MHz, CD_2Cl_2): δ = 5.08 ppm (t, J = 5.52 Hz, C*P*Ph_3), -0.93 ppm (s, Os*P*Ph_3). ^{13}C-NMR (100.6 MHz, CD_2Cl_2, plus ^{13}C-dept 135, ^{1}H-^{13}C HSQC and ^{1}H-^{13}C HMBC): δ = 317.1 (s, C^1), 214.6 (s, C^7), 172.6 (s, C^5), 169.5 (s, C^6), 168.6 (s, C^4), 149.7 (s, C^3), 70.96 (s, C^8), 70.66 (s, C^9), 148.6-119.5 ppm (other aromatic carbons, C^{10}, and C^{11}). HRMS (ESI): m/z calcd for $[C_{77}H_{59}ClN_2OOsP_3]^+$: 1347.3135, found: 1347.3127; Elemental analysis calcd (%) for $C_{77}H_{59}Cl_2N_2OOsP_3$: C 66.90, H 4.30, found: C 70.15, H 4.64.

Synthesis of DPC

The carbolong complex S1 was synthesized according to published procedures[16]. Excess acid HCl • Et_2O (1 mL of a 2 M solution in ether) was added to the DCM solution (10 mL) of carbolong complex S1 (0.20 g, 0.17 mmol) and 3,8-bis(ethynyl)-1,10-phenanthroline Phen-D1 (0.018 g, 0.08 mmol) using standard Schlenk techniques. The reaction mixture was stirred at room temperature for 4 h to yield a dark red solution. The solution was evaporated under vacuum to a volume of approximately 1 mL and then washed with Et_2O (1× 20 mL) to afford a violet solid. The solid was purified by flash chromatography on silica gel (eluent: 20:1 DCM/MeOH) to yield complex 2 as a dark red solid. Yield: 0.19 g, 91%. ^{1}H-NMR (400.1 MHz, CD_2Cl_2): δ = 8.79 (s, 2H), 5.94 (d, *J* = 16.29 Hz, 2H, $C^{11}H$), 5.11 (s, 4H, $C^{9}H$), 4.42 (s, 4H, $C^{8}H$), 7.87-6.97 (100H, other aromatic protons, $C^{3}H$, and $C^{12}H$). ^{31}P-NMR (162.0 MHz, CD_2Cl_2): δ = 5.04 ppm (t, *J* = 4.21 Hz, C*P*Ph_3), -0.88 ppm (d, *J* = 4.70 Hz, Os*P*Ph_3). ^{13}C-NMR (100.6 MHz, CD_2Cl_2, plus ^{13}C-dept 135, ^{1}H-^{13}C HSQC and ^{1}H-^{13}C HMBC): δ = 317.4 (s, C^1), 215.9 (s, C^7), 172.7 (s, C^5), 169.2 (s, C^6), 168.4 (d, *J* = 22.81 Hz, C^4), 150.2 (s, C^3), 70.90 (s, C^8), 70.66 (s, C^9), 147.8-119.6 ppm (other aromatic carbons, C^{10}, and C^{11}). HRMS (ESI): m/z calcd for $[C_{142}H_{110}Cl_2N_2O_2Os_2P_6]^{2+}$: 1256.7791, found: 1256.7783. Elemental analysis calcd (%) for $C_{142}H_{110}Cl_4N_2O_2Os_2P_6$: C 65.99, H 4.29, found: C 65.68, H 4.43.

SUPPLEMENTARY REFERENCES

1. Lin Y, et al. An Electron Acceptor Challenging Fullerenes for Efficient Polymer Solar Cells. *Adv. Mater.* **27**, 1170-1174 (2015).

2. Li W, Ye L, Li S, Yao H, Ade H, Hou J. A High-Efficiency Organic Solar Cell Enabled by the Strong Intramolecular Electron Push–Pull Effect of the Nonfullerene Acceptor. *Adv. Mater.* **30**, 1707170 (2018).

3. Yuan J, et al. Enabling low voltage losses and high photocurrent in fullerene-free organic photovoltaics. *Nat. Commun.* **10**, 570 (2019).

4. Wang R, et al. Rational Tuning of Molecular Interaction and Energy Level Alignment Enables High-Performance Organic Photovoltaics. *Adv. Mater.* **31**, 1904215 (2019).

5. Yuan J, et al. Fused Benzothiadiazole: A Building Block for n-Type Organic Acceptor to Achieve High-Performance Organic Solar Cells. *Adv. Mater.* **31**, 1807577 (2019).

6. Cui Y, et al. Single-Junction Organic Photovoltaic Cells with Approaching 18% Efficiency. *Adv. Mater.* **32**, 1908205 (2020).

7. Yuan J, et al. Single-Junction Organic Solar Cell with over 15% Efficiency Using Fused-Ring Acceptor with Electron-Deficient Core. Joule **3**, 1140-1151 (2019).

8. Zhu L, et al. Single-junction organic solar cells with over 19% efficiency enabled by a refined double-fibril network morphology. *Nat. Mater.* **21**, 656-663 (2022).

9. Qin Y, et al. 18.4% efficiency achieved by the cathode interface engineering in non-fullerene polymer solar cells. *Nano Today* **41**, 101289 (2021).

10. Gong Y, et al. Water-Induced Formation of α-MoO3 Microcrystals as Anode Buffer Layer for Highly Efficient Polymer Solar Cells. *Energy Technol Ger* **9**, 2100718 (2021).

11. Yao J, et al. Cathode engineering with perylene-diimide interlayer enabling over 17% efficiency single-junction organic solar cells. *Nat. Commun.* **11**, 2726 (2020).

12. Wang Y, et al. Synthesis of N,S-Doped Carbon Quantum Dots for Use in Organic Solar Cells as the ZnO Modifier To Eliminate the Light-Soaking Effect. *Acs Appl. Mater. Inter.* **11**, 2243-2253 (2019).

13. Tran V-H, Eom SH, Yoon SC, Kim S-K, Lee S-H. Enhancing device performance of inverted organic solar cells with SnO2/Cs2CO3 as dual electron transport layers. *Org. Electron* **68**, 85-95 (2019).

14. Chen G, et al. Efficient Cathode Buffer Material Based on Dibenzothiophene-S,S-dioxide for Both Conventional and Inverted Organic Solar Cells. *ACS Omega* **7**, 38613-38621 (2022).

15. Yang R, et al. Oriented Quasi-2D Perovskites for High Performance Optoelectronic Devices. *Nat. Mater.* **30**, 1804771 (2018).

16. Zhuo, Q. *et al.* Multiyne chains chelating osmium via three metal-carbon sigma bonds. *Nat. Commun.* **8**, 1912 (2017).
