# Supplementary material for: Phenanthroline-carbolong interface suppress chemical interactions with active layer enabling long-time stable organic solar cells
Source: Nat Commun. 2023 Jun 16;14:3571. doi: 10.1038/s41467-023-39223-9 (PMC10272153; doi:10.1038/s41467-023-39223-9)
Supplement: Supplementary file 3 — Description of Additional Supplementary Files [file 41467_2023_39223_MOESM3_ESM.pdf]

File name: Supplementary Data 1

Description: Single crystal structure of SPC.
