# Supplementary material for: Phenanthroline-carbolong interface suppress chemical interactions with active layer enabling long-time stable organic solar cells
Source: Nat Commun. 2023 Jun 16;14:3571. doi: 10.1038/s41467-023-39223-9 (PMC10272153; doi:10.1038/s41467-023-39223-9)
Supplement: Supplementary file 5 — Solar Cells Reporting Summary [file 41467_2023_39223_MOESM5_ESM.pdf]

nature research

Corresponding author(s): Haiping Xia, Feng He

Solar Cells Reporting Summary

Nature Research wishes to improve the reproducibility of the work that we publish. This form is intended for publication with all accepted papers reporting the characterization of photovoltaic devices and provides structure for consistency and transparency in reporting. Some list items might not apply to an individual manuscript, but all fields must be completed for clarity.

For further information on Nature Research policies, including our data availability policy, see Authors & Referees.

Experimental design

Please check: are the following details reported in the manuscript?

1. Dimensions

Area of the tested solar cells	☒ Yes ☐ No	Relative information is provided in method section.
Method used to determine the device area	☒ Yes ☐ No	Relative information is provided in method section.

2. Current-voltage characterization

Current density-voltage (J-V) plots in both forward and backward direction	☐ Yes ☒ No	J-V plot in forward direction since there is no hysteresis in polymer solar cells.
Voltage scan conditions<br>*For instance: scan direction, speed, dwell times*	☒ Yes ☐ No	he voltage range for single junction OSC is from -0.2 V to 1 V, and for tandem solar cell is from -0.2 V to 2.5 V. The voltage step and delay time were 10 mV and 1 ms, respectively.
Test environment<br>*For instance: characterization temperature, in air or in glove box*	☒ Yes ☐ No	Devices were characterized at room temperature in In N2-filled glove box.
Protocol for preconditioning of the device before its characterization	☐ Yes ☒ No	No preconditioning protocol.
Stability of the J-V characteristic<br>*Verified with time evolution of the maximum power point or with the photocurrent at maximum power point; see ref. 7 for details.*	☐ Yes ☒ No	Organic photovoltaic devices show no decay or instability during the test of J-V characteristics. The MPP output is consistent with the J-V curve.

3. Hysteresis or any other unusual behaviour

Description of the unusual behaviour observed during the characterization	☐ Yes ☒ No	No hysteresis was observed in our device.
Related experimental data	☐ Yes ☒ No	No.

4. Efficiency

External quantum efficiency (EQE) or incident photons to current efficiency (IPCE)	☒ Yes ☐ No	Provided in Figures 3c and 6d.
A comparison between the integrated response under the standard reference spectrum and the response measure under the simulator	☒ Yes ☐ No	The difference between the integrated current from EQE and the short-circuit current from J-V curve measured under AM 1.5G simulated sunlight is within 3% difference which is within the accuracy confidence of the measurements.
For tandem solar cells, the bias illumination and bias voltage used for each subcell	☒ Yes ☐ No	During EQE measurement for tandem device, light bias with 550 nm short pass fiter and 800nm long pass filter were applied seperately to obtain rear cell and front cell EQE value. No additional illumination and voltage bias were applied during measurements.

5. Calibration

Light source and reference cell or sensor used for the characterization	☒ Yes ☐ No	Relative information is provided in method section.

November 2017

Confirmation that the reference cell was calibrated and certified

☒ Yes ☐ No — Relative information is provided in method section.

Calculation of spectral mismatch between the reference cell and the devices under test

☒ Yes ☐ No — Relative information is provided in method section.

6. Mask/aperture

Size of the mask/aperture used during testing

☒ Yes ☐ No — Device area is determined by a mask with area of 0.046 cm2

Variation of the measured short-circuit current density with the mask/aperture area

☐ Yes ☒ No — The variation is within 0.3%.

7. Performance certification

Identity of the independent certification laboratory that confirmed the photovoltaic performance

☐ Yes ☒ No — The photovoltaic performance of our devices was not confirmed from independent certification laboratories

A copy of any certificate(s)
Provide in Supplementary Information

☐ Yes — *State where this information can be found in the text.*
☐ No — *Explain why this information is not reported/not relevant.*

8. Statistics

Number of solar cells tested

☒ Yes ☐ No — The average PCE of OSC is obtained from at least 10 different cells and of tandem cells is obtained from 30 cells

Statistical analysis of the device performance

☒ Yes ☐ No — Provided in Table 2 and Figure 6e.

9. Long-term stability analysis

Type of analysis, bias conditions and environmental conditions
For instance: illumination type, temperature, atmosphere humidity, encapsulation method, preconditioning temperature

☒ Yes ☐ No — Relative information is provided in method section.
